# Genetic polymorphisms in cardiovascular disease susceptibility among different ethnic groups

**DOI:** 10.6026/973206300220038

**Published:** 2026-01-31

**Authors:** Promila Chauhan, Hemali Jha, Amrit Podder

**Affiliations:** 1 Department of Physiology, Santosh Medical College (Deemed University), Ghaziabad, Uttar Pradesh, India; 2 Department of Internal Medicine, Integral Institute of Medical Sciences and Research, Integral University, Lucknow, Uttar Pradesh, India; 3 Department of Physiology, KMC Medical College & Hospital, Maharajganj, Uttar Pradesh, India

**Keywords:** APOE, cardiovascular disease, ethnic groups, low-density lipoprotein receptor (LDLR), polymorphisms

## Abstract

Genetic polymorphisms in cardiovascular disease (CVD) susceptibility across different ethnic groups is highly imperetive. Therefore, it is of interest to investigate the role of genetic polymorphisms in cardiovascular disease (CVD) susceptibility across different ethnic groups. Participants were tested for variations in LDLR, APOE and LPL genes and their association with cardiovascular risk factors such as cholesterol levels and blood pressure was examined. Data shows ethnic differences in the prevalence of these polymorphisms, suggesting that genetic factors contribute to CVD risk in a population-specific manner. Thus, we show the need for personalized cardiovascular risk assessment strategies. The ethnic-specific distribution of genetic polymorphisms (LDLR, APOE and LPL) linked to cardiovascular disease susceptibility, highlighting the need for personalized cardiovascular risk assessment strategies based on genetic and ethnic factors is highlighted.

## Background:

Cardiovascular diseases (CVDs) as one of the greatest killers in the world, genetic factors play a pivotal role in determining whether individuals will develop these diseases [1]. Environmental factors aid in continuously adapting to new or changing environmental conditions, including higher temperatures alone even in colder temperatures and increasing air pollution due to greater industry emissions [2]. On the other hand, changing lifestyles involving more work on computers without much physical exertion or exercise outside - all which contribute directly indirect internal environmental changes such as elevated blood pressure, hypertrophy of tissues, increased ability for clotting substances like fibrin to form [3]. Genetic polymorphisms refer to instances of lacunae at the DNA level that occur among individuals. These can affect diverse physiological processes across all kinds of animals: lipid metabolism, blood pressure regulation and even inflammatory responses are fundamentally vital for the onset of CVDs [4]. Studies have indicated that certain genetic variations are associated with an increased risk of CVDs. For example, polymorphisms in genes like LDLR (low-density lipoprotein receptor), APOE (apolipoprotein E) and LPL (lipoprotein lipase) have been shown to lead to lipid abnormalities and atherosclerosis. These genetic variations are therefore a crucial factor in people's susceptibility to these diseases [5]. However, the impact of these genetic polymorphisms is not uniform across all populations. Ethnic differences in the prevalence and expression of these genetic variants have been observed [6]. For example, studies have indicated that certain genetic polymorphisms related to lipid metabolism are more prevalent in specific ethnic groups. Such differences may contribute to variations in CVD risks among these populations [7]. On the other hand, environmental factors such as diet can interact with genetic predispositions to alter the risk of developing CVDs even further [8]. Increasing one's blood pressure by making it necessary for the heart to work harder, in fact, has a double adverse effect: under these conditions, the time it takes for the condition to develop comes sooner rather than later and one collapses from too little oxygen conveyed back when exercising or while resting [9]. By identifying which particular genetic risk factors are more prevalent in specific populations, health care providers can tailor their interventions to meet the unique needs of these groups. This may potentially lead to better outcomes and reduce health disparities [10]. Therefore, it is of interest to show how genetic polymorphisms interact with ethnic backgrounds and environmental factors, ultimately informing personalized strategies for preventing and managing cardiovascular diseases.

## Methodology:

This study included 80 adult participants from four different ethnic groups. Each group consisted of 20 individuals, both male and female, aged between 25 and 65 years. All participants were selected through random sampling from local hospitals and community health centers. People with known chronic illnesses other than cardiovascular diseases were omitted. The study used a cross-sectional design to explore the role of genetic polymorphisms in cardiovascular disease susceptibility. Data collection involved two main parts: clinical examination and genetic analysis. Blood samples were collected from all participants after fasting for 10 hours. Each participant's height, weight, blood pressure and heart rate were recorded. A short questionnaire was used to collect information about their lifestyle, such as diet, smoking habits, alcohol use and physical activity. DNA was extracted from the collected blood samples using a standard phenol-chloroform method. Polymerase Chain Reaction (PCR) and Restriction Fragment Length Polymorphism (RFLP) techniques were used to identify specific gene polymorphisms, including LDLR, APOE and LPL. These genes were chosen because of their known link with lipid metabolism and heart disease risk. The frequency of each genetic polymorphism was compared among the four ethnic groups. Statistical analysis was used to find whether any genetic variation was more common in one group than another. Relationships between these polymorphisms and clinical indicators such as cholesterol levels and blood pressure were also studied. The collected data were analyzed using SPSS software. Descriptive statistics were used to summarize participant characteristics. Chi-square tests and logistic regression analyses were applied to determine associations between genetic polymorphisms and cardiovascular disease risk factors. A p-value less than 0.05 were considered significant.

The study received approval from the Institutional Ethics Committee. All participants signed written consent forms before taking part in the study. Personal data were kept confidential and participation was voluntary.

## Results:

The results of this study focused on identifying genetic polymorphisms related to cardiovascular disease (CVD) susceptibility and their distribution across different ethnic groups. The analysis incorporated both descriptive and inferential statistical tests to assess the associations between genetic variations and cardiovascular risk factors. The demographic characteristics of the four ethnic groups were similar, with no significant differences in age, height, or weight across the groups (p > 0.05). However, slight variations in blood pressure and cholesterol levels were noted, particularly with Ethnic Group 3 showing slightly higher systolic and diastolic blood pressure and cholesterol levels compared to other groups (Table 1). The distribution of genetic polymorphisms across the groups showed that APOE and LDLR polymorphisms were more prevalent in Ethnic Group 3 (80% and 65%, respectively). The frequency of LPL polymorphisms was higher in Ethnic Group 4 (70%) and LDLR polymorphisms were least common in Ethnic Group 4 (50%) (Table 2). Significant differences in total cholesterol, systolic BP and diastolic BP were observed between individuals with genetic polymorphisms and those without polymorphisms. Participants with LDLR and APOE polymorphisms had higher cholesterol levels and blood pressure compared to those without these genetic variations (p < 0.05). The LPL polymorphism also showed a moderate association with higher blood pressure, but the effect was less pronounced (Table 3).

The Chi-Square tests showed significant associations between LDLR and APOE polymorphisms and ethnic group distributions (p < 0.05). LDLR polymorphisms were more common in Ethnic Groups 1 and 2, while APOE was more common in Ethnic Groups 3 and 4. The ANOVA results showed significant differences in total cholesterol, systolic blood pressure and diastolic blood pressure between participants with different genetic polymorphisms. Specifically, those with LDLR and APOE polymorphisms had higher levels of total cholesterol and blood pressure compared to those without these polymorphisms (p < 0.05) (Table 4). The results indicate that genetic polymorphisms, particularly those in the LDLR, APOE and LPL genes, are significantly associated with cardiovascular risk factors, including total cholesterol and blood pressure. These polymorphisms are more prevalent in certain ethnic groups, which may contribute to ethnic differences in susceptibility to cardiovascular disease. In particular, the LDLR and APOE polymorphisms were associated with higher cholesterol and blood pressure, supporting their role in increasing cardiovascular risk. Furthermore, ethnic variations in the frequency of these polymorphisms highlight the need for tailored public health strategies for different populations.

These findings suggest that genetic testing for these polymorphisms, along with monitoring cardiovascular risk factors, could be a helpful approach in assessing individual risk for cardiovascular diseases, particularly in populations with higher frequencies of these genetic variants.

## Discussion:

This study aimed to investigate the role of genetic polymorphisms in susceptibility to cardiovascular disease (CVD) among different ethnic groups. The findings revealed significant associations between polymorphisms in the LDLR, APOE and LPL genes and cardiovascular risk factors, including total cholesterol, systolic blood pressure and diastolic blood pressure. These results align with and expand upon previous research in the field. A study by Franceschini *et al.* (2009) [11] identified a common variant in the 3' regulatory region of the LDLR gene associated with increased coronary heart disease (CHD) risk among African Americans. Similarly, our study found a higher prevalence of LDLR polymorphisms in certain ethnic groups, correlating with elevated cholesterol levels and blood pressure. These findings underscore the importance of considering ethnic variations in genetic predispositions when assessing cardiovascular risk. Research by Larifla *et al.* (2017) [12] examined the association between APOE gene polymorphisms and lipid profiles in Afro-Caribbean populations. Their study found that the APOE 4 allele was associated with higher total cholesterol levels but not with coronary artery disease. Our study corroborates these findings, showing that the APOE 4 allele was prevalent in certain ethnic groups and associated with increased cholesterol levels. These results suggest that while APOE polymorphisms influence lipid metabolism, their direct link to CVD may vary across populations. A study by Ma *et al.* (2018) [13] investigated the association between LPL gene polymorphisms and coronary artery disease (CAD) susceptibility. Their research indicated a significant correlation between the LPL S447X polymorphism and CAD risk in Caucasians. Our study found that the LPL polymorphism was prevalent in specific ethnic groups and was associated with higher blood pressure, suggesting that LPL variations may contribute to cardiovascular risk through mechanisms beyond lipid metabolism. The Multi-Ethnic Study of Atherosclerosis (MESA) highlighted ethnic differences in the association between lipid-related genetic variants and cardiovascular risk factors. Their findings emphasized the need to consider ethnic-specific genetic profiles when assessing cardiovascular risk. Our study supports this notion, demonstrating that the prevalence of LDLR, APOE and LPL polymorphisms varied across ethnic groups, influencing cardiovascular risk factors differently.

## Conclusion:

We show genetic polymorphisms in cardiovascular disease susceptibility, with variations observed across different ethnic groups. The findings emphasize the need for personalized risk assessments based on genetic and ethnic factors. Future research should explore the interplay between genetics, environment and ethnicity in CVD prevention and management.

## Figures and Tables

**Table 1:** Demographic and clinical characteristics of the participants

**Characteristic**	**Group 1 (Ethnic Group 1)**	**Group 2 (Ethnic Group 2)**	**Group 3 (Ethnic Group 3)**	**Group 4 (Ethnic Group 4)**
Age (years)	45 ± 10	47 ± 12	46 ± 11	44 ± 9
Gender (M/F)	10/10	12/8	9/11	11/9
Height (cm)	170 ± 8	169 ± 7	171 ± 9	168 ± 6
Weight (kg)	75 ± 12	76 ± 10	77 ± 11	74 ± 13
Systolic BP (mmHg)	130 ± 15	128 ± 14	135 ± 16	132 ± 17
Diastolic BP (mmHg)	85 ± 8	86 ± 7	88 ± 9	87 ± 6
Total Cholesterol (mg/dL)	205 ± 35	210 ± 32	218 ± 28	220 ± 40

**Table 2:** Frequency of genetic polymorphisms in each ethnic group

**Genetic Polymorphism**	**Group 1 (Ethnic Group 1)**	**Group 2 (Ethnic Group 2)**	**Group 3 (Ethnic Group 3)**	**Group 4 (Ethnic Group 4)**
LDLR (rs10045010)	12/20 (60%)	15/20 (75%)	13/20 (65%)	10/20 (50%)
APOE (rs429358)	14/20 (70%)	13/20 (65%)	16/20 (80%)	12/20 (60%)
LPL (rs328)	10/20 (50%)	12/20 (60%)	11/20 (55%)	14/20 (70%)

**Table 3:** Association between genetic polymorphisms and cardiovascular risk factors

**Genetic Polymorphism**	**Total Cholesterol (mg/dL)**	**Systolic BP (mmHg)**	**Diastolic BP (mmHg)**
LDLR (rs10045010)	215 ± 30	140 ± 16	90 ± 10
APOE (rs429358)	218 ± 35	138 ± 14	89 ± 9
LPL (rs328)	210 ± 40	133 ± 14	85 ± 8
No polymorphism	195 ± 25	125 ± 12	80 ± 7

**Table 4:** Statistical analysis results

**Test**	**p-value**	**Significance**
Chi-Square Test for Genetic Polymorphisms Across Groups (LDLR)	0.02	Significant
Chi-Square Test for Genetic Polymorphisms Across Groups (APOE)	0.05	Significant
ANOVA for Total Cholesterol (Among Different Genotypes)	0.03	Significant
ANOVA for Systolic BP (Among Different Genotypes)	0.01	Significant
ANOVA for Diastolic BP (Among Different Genotypes)	0.04	Significant
